# Inhibiting the IRE1*α* Axis of the Unfolded Protein Response Enhances the Antitumor Effect of AZD1775 in *TP53* Mutant Ovarian Cancer

**DOI:** 10.1002/advs.202105469

**Published:** 2022-05-26

**Authors:** Rourou Xiao, Lixin You, Li Zhang, Xichen Guo, Ensong Guo, Faming Zhao, Bin Yang, Xi Li, Yu Fu, Funian Lu, Zizhuo Wang, Chen Liu, Wenju Peng, Wenting Li, Xiaohang Yang, Yingyu Dou, Jingbo Liu, Wei Wang, Tianyu Qin, Yaoyuan Cui, Xiaoxiao Zhang, Fuxia Li, Yang Jin, Qingping Zeng, Beibei Wang, Gordon B. Mills, Gang Chen, Xia Sheng, Chaoyang Sun

**Affiliations:** ^1^ National Clinical Research Center for Gynecology and Obstetrics Tongji Hospital Tongji Medical College Huazhong University of Science and Technology Wuhan 430030 China; ^2^ Cancer Biology Research Center Tongji Hospital Tongji Medical College Huazhong University of Science and Technology Wuhan 430030 China; ^3^ Department of Gynecology and Obstetrics, Tongji Hospital Tongji Medical College Huazhong University of Science and Technology Wuhan 430030 China; ^4^ Key Laboratory of Environment and Health Ministry of Education & Ministry of Environmental Protection and State Key Laboratory of Environmental Health (Incubation) School of Public Health Tongji Medical College Huazhong University of Science and Technology Wuhan 430030 China; ^5^ Department of Obstetrics and Gynecology The First Affiliated Hospital of Zhengzhou University Zheng Zhou 450052 China; ^6^ Department of gynecology First Affiliated Hospital Shihezi University School of Medicine Shihezi Xinjiang 832000 P. R. China; ^7^ Department of Biosciences University of Oslo Oslo 0371 Norway; ^8^ Fosun Orinove Inc. Unit 211, Building A4, 218 Xinhu Street Suzhou 215000 China; ^9^ Department of Cell Development and Cancer Biology Knight Cancer Institute Oregon Health and Sciences University Portland OR 97201 USA

**Keywords:** AZD1775, mutant TP53, ovarian cancer, UPR, WEE1

## Abstract

Targeting the G2/M checkpoint mediator WEE1 has been explored as a novel treatment strategy in ovarian cancer, but mechanisms underlying its efficacy and resistance remains to be understood. Here, it is demonstrated that the WEE1 inhibitor AZD1775 induces endoplasmic reticulum stress and activates the protein kinase RNA‐like ER kinase (PERK) and inositol‐required enzyme 1*α* (IRE1*α*) branches of the unfolded protein response (UPR) in *TP53* mutant (mt*TP53*) ovarian cancer models. This is facilitated through NF‐*κ*B mediated senescence‐associated secretory phenotype. Upon AZD1775 treatment, activated PERK promotes apoptotic signaling via C/EBP‐homologous protein (CHOP), while IRE1*α*‐induced splicing of XBP1 (XBP1s) maintains cell survival by repressing apoptosis. This leads to an encouraging synergistic antitumor effect of combining AZD1775 and an IRE1*α* inhibitor MKC8866 in multiple cell lines and preclinical models of ovarian cancers. Taken together, the data reveal an important dual role of the UPR signaling network in mt*TP53* ovarian cancer models in response to AZD1775 and suggest that inhibition of the IRE1*α*‐XBP1s pathway may enhance the efficacy of AZD1775 in the clinics.

## Graphical abstract

WEE1 inhibitor AZD1775 disrupts endoplasmic reticulum proteostasis and activates the protein kinase RNA‐like ER kinase and inositol‐required enzyme 1*α* (IRE1*α*) arms of the unfolded protein response in ovarian cancer cells with mutant p53, which is mediated by NF‐*κ*B‐induced senescence‐associated secretory phenotype. Cotargeting of WEE1 and IRE1*α*‐XBP1s pathway may be synergistic in advanced ovarian cancer with mutant p53.

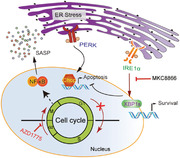

## 1 Introduction

Ovarian cancer is a malignant tumor of the female reproductive system with poor prognosis. High‐grade serous ovarian cancer (HGSOC) is the most common type of ovarian cancer with *TP53* mutation as a *sine qua non*.^[^ ^1^ ^]^ P53 (encoded by *TP53*) is fundamental to regulating the G1/S checkpoint, whose mutation leaves the cells largely dependent on a functional G2/M checkpoint for DNA repair, wherein WEE1 kinase plays a key role.^[^ ^2^, ^3^ ^]^ Thus, targeting WEE1 to disrupt the G2 checkpoint accelerates cellular progression, leading to mitotic catastrophe and subsequent cell death.^[^ ^4^, ^5^ ^]^ AZD1775, a selective small molecule inhibitor of WEE1 kinase, has shown promising antitumor activity in patients with refractory or platinum‐resistant ovarian cancer,^[^ ^6^ ^]^ especially those with *TP53* mutations.^[^ ^7^, ^8^, ^9^ ^]^ However, mechanistic insights into its efficacy and resistance in ovarian cancer remains to be explored.

The unfolded protein response (UPR) is an adaptive mechanism in response to proteostatic stress in the endoplasmic reticulum (ER).^[^ ^10^ ^]^ It consists of three signaling pathways respectively initiated by the ER stress sensors: inositol‐required enzyme 1*α* (IRE1*α*), protein kinase RNA‐like ER kinase (PERK), and activating transcription factor 6 (ATF6).^[^ ^10^ ^]^ Upon ER stress, X‐Box‐binding protein 1 (XBP1) mRNA is spliced by activated IRE1*α* to generate a potent transcription factor XBP1s, which regulate the expression of numerous genes involved in protein folding, quality control, and lipid synthesis.^[^ ^11^ ^]^ Activated PERK transiently reduces global protein synthesis through eukaryotic translation initiation factor 2 subunit alpha (eIF2*α*) phosphorylation, but activates C/EBP‐homologous protein (CHOP) to trigger apoptosis upon irremediable stress.^[^ ^12^ ^]^ ATF6 is cleaved and activated at the Golgi, and regulates the expression of genes involved in protein folding and degradation.^[^ ^13^ ^]^A series of recent studies have unveiled important roles for UPR, particularly the PERK and IRE1*α* arms, in cancer cell survival, apoptosis, and response to therapy.^[^ ^14^ ^]^ Small molecules targeting these stress response pathways have shown promising efficacy in different cancer models and are now under rapid translational development.^[^ ^10^ ^]^ For instance, the IRE1*α* RNase inhibitor MKC8866 disrupted the interaction between the IRE1*α*‐XBP1s and c‐Myc oncogenic programs in prostate and breast cancer models and is proposed as a novel therapy for Myc‐hyperactivated tumors.^[^ ^15^, ^16^, ^17^ ^]^

Although UPR has been implicated in therapeutic responsiveness, little is known about whether and how it is involved in response to cell cycle‐directed therapy. In this study, we demonstrate that UPR is induced by WEE1 inhibitor AZD1775 in ovarian cancer models with mutant *TP53*. Disrupted ER proteostasis and UPR activation is resultant of NF‐*κ*B‐mediated senescence‐associated secretory phenotype (SASP). Consistently, AZD1775 response is significantly associated with senescence as well as UPR pathway activity in a number of ovarian cancer patient datasets, supporting the clinical relevance between these pathways. More importantly, PERK facilitates apoptosis by activating CHOP after AZD1775 exposure, whereas the IRE1*α*‐XBP1s axis promotes survival by repressing apoptosis. Furthermore, combination of AZD1775 and the IRE1*α* inhibitor MKC8866 show remarkable synergistic effect in multiple *TP53*‐mutant cell lines and patient‐derived xenograft (PDX) models of ovarian cancer. Therefore, our study uncovers an important dual role of the UPR network in response to AZD1775 treatment in *TP53*‐mutated ovarian cancer, and provide a rationale for targeting the IRE1*α*‐XBP1s axis to enhance the efficacy of AZD1775.

## 2 Results

### 2.1 AZD1775 Triggers UPR in *TP53* Mutant Ovarian Cancer Cells

**Figure 1:**
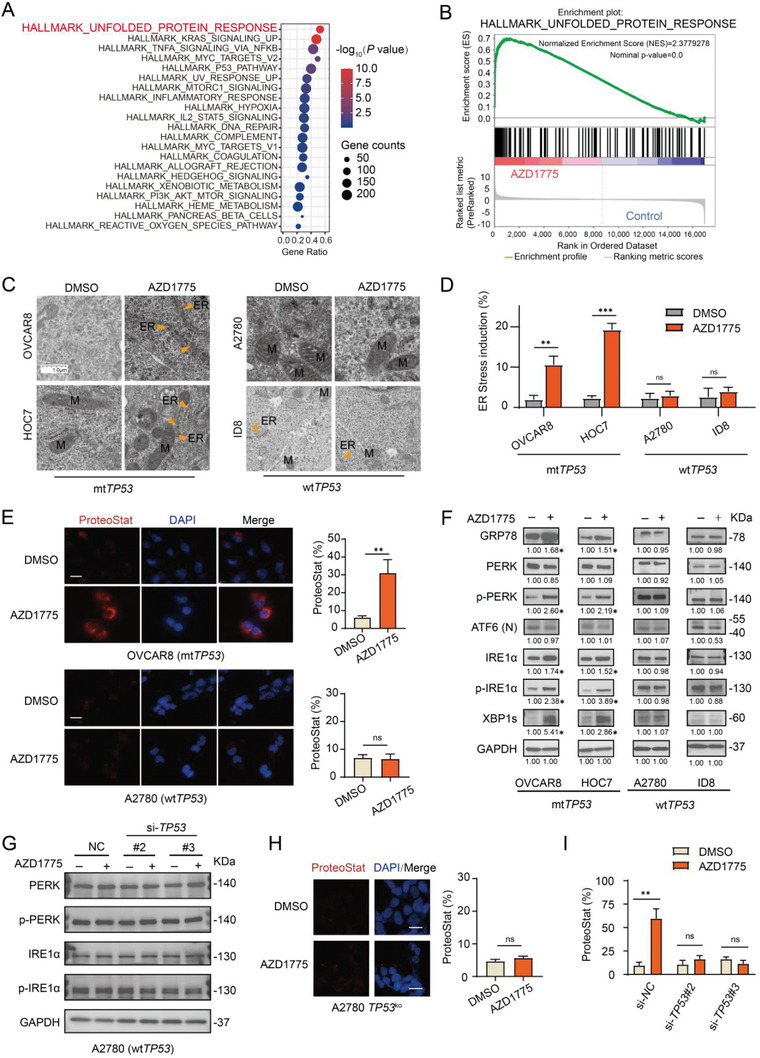
AZD1775 triggers UPR in *mtTP53* ovarian cancer cells. A) Hallmark pathway enriched by GSEA. OVCAR8 cells were treated with DMSO or 400 × 10^−9^ m AZD1775 for 48 h and subjected to RNA‐seq analysis. B) GSEA plot of the Hallmark pathway UNFOLDED PROTEIN RESPONSE enriched by AZD1775 treatment. C) Representative images by electron microscopy of the ER (indicated by the orange arrows). The cells were treated with DMSO or 400 × 10^−9^ m AZD1775 for 48 h. Scale bar, 1.0 µm. D) Quantification of the percentage of cells with expanded volume or increased number of ER in (C). Error bars represent the standard deviation (SD) of the mean (*n* = 3), **p < 0.01, ***p < 0.001, ns, not significant, as determined by the unpaired two‐tailed Student's *t*‐test. E) Representative images and quantification of aggregated protein levels. Cells were treated with DMSO or 400 × 10^−9^ m AZD1775 for 48 h and protein aggregates were detected using the Proteostat Dye. Error bars represent the SD of the mean (*n* = 3), **p < 0.01, ns, not significant, as determined by the unpaired two‐tailed Student's *t*‐test. Scale bar, 20 µm. F) Western blot analysis of the protein expression levels of the key proteins in the UPR. Ovarian cancer cell lines were treated with or without 400 × 10^−9^ m AZD1775 for 72 h. The data represent three independent experiments. The numbers represent the mean quantification (*n* = 3) of the gray scale using image Lab software 6.0.1. after normalizing to GAPDH, *p < 0.05, as determined by the unpaired two‐tailed Student's *t*‐test. G) Western blot analysis of the protein expression levels of PERK, p‐PERK, IRE1*α*, p‐IRE1*α*. A2780 cells were treated with or without 400 × 10^−9^ m AZD1775 for 72 h after transfected with scramble siRNA (NC) or *TP53* siRNA. #2 and #3 denote different *TP53* siRNAs. H) Representative images and quantification of aggregated proteins. A2780‐*TP53* ^ko^@9 clone cells were treated with DMSO or 400 × 10^−9^ m AZD1775 for 48 h and stained with Proteostat Dye. The data represent three independent experiments. Scale bar, 20 µm. Error bars represent the SD of the mean (*n* = 3), ns, not significant, as determined by the unpaired two‐tailed Student's *t*‐test. I) quantification of aggregated proteins in Figure S1K (Supporting information). OVCAR8 cells were treated with or without 400 × 10^−9^ m AZD1775 for 72 h after transfected with scramble siRNA (NC) or *TP53* siRNAs. The data represent three independent experiments. Scale bar, 20 µm. Error bars represent the SD of the mean (*n* = 3), **p < 0.01, ns, not significant, as determined by the unpaired two‐tailed Student's *t*‐test.

To better understand the molecular biology of WEE1 inhibition in ovarian cancer, we performed RNA sequencing (RNA‐seq) analysis using OVCAR8 cells treated with AZD1775. The proper inhibition of WEE1 activity by AZD1775 was reflected by decreased phosphorylation of its downstream substrate cyclin‐dependent kinase 1 (CDK1) (Tyr15) and increased *γ*‐H2AX (Figure S1A, Supporting Information). Differential gene expression analysis revealed 1064 genes whose expression was significantly altered by AZD1775 compared to control (determined by adjusted *p* value), in which 491 genes were upregulated (Figure S1B, Supporting Information). The markedly repressed Hallmark G2/M checkpoint pathway activity was confirmed by gene set enrichment analysis (GSEA) (Figure S1C, Supporting Information). Of interest, the UPR pathway ranked first among all the significantly enriched Hallmark pathways according to *p* value and gene ratio (**Figure** **1**A,B), suggesting that AZD1775 may induce ER stress in these cells. This was validated by the enrichment of multiple ER and UPR related Gene Ontology (GO) pathways, including the PERK‐mediated UPR pathway and intrinsic apoptotic signaling downstream of ER stress (Figure S1D, Supporting Information).

To assess the presence of ER stress with an orthogonal approach, we applied transmission electron microscopy to examine the changes in ER ultrastructure using four ovarian cancer cell lines with different *TP53* state (human A2780 and murine ID8 are *TP53* wild‐type ovarian cancer cell lines, while human OVCAR8 and HOC7 harbor mutant *TP53*). The AZD1775 concentration was determined based on the markedly reduced p‐CDK1 (Tyr15) and induced *γ*‐H2AX (Figure S1E, Supporting Information). The volume and number of ER were significantly increased in *TP53* mutant (mt*TP53*) OVCAR8 and HOC7 cells treated with AZD1775, but not in A2780 and ID8 cells with wild‐type TP53 (wt*TP53*) (Figure 1C,D). Consistently, aggregated proteins were observed in OVCAR8 cells by proteostat dye, while not in A2780 cells (Figure 1E).

Next, we examined the activation of the three UPR pathways. Western blot analyses observed significantly elevated expression of glucose‐regulated protein 78 (GRP78), p‐PERK, p‐IRE1*α*, and XBP1s in AZD1775‐treated OVCAR8 and HOC7 cells, while cleaved ATF6 level was not altered (Figure 1F). In line with these results, siRNA mediated WEE1 knockdown (Figure S1F, Supporting Information) induced a similar activation pattern of the UPR pathways (Figure S1G, Supporting Information). In contrast, little to no change was seen in the expression of these proteins in A2780 and ID8 cells (Figure 1F). These data demonstrate that AZD1775 induces ER stress and activates the PERK and IRE1*α* arms of UPR in mt*TP53* ovarian cancer cells.

To further clarify the role of *TP53* state in UPR induction, we performed siRNA mediated *TP53* knockdown in A2780 cells (Figure S1H, Supporting Information). Surprisingly, loss of *TP53 through* knockdown by siRNA did not significantly activate AZD1775‐induced UPR pathway in A2780 cells with wt*TP53* (Figure 1G). Furthermore, CRISPR‐Cas9 mediated *TP53* knockout (*TP53* ^ko^) (Figure S1I, Supporting Information) also showed little to no increase in aggregated proteins (Figure 1H). Notably, *TP53* knockdown significantly abrogated the activation of UPR pathway (Figure S1J, Supporting Information) and erased the protein aggregation (Figure 1I; and Figure S1K, Supporting Information) induced by AZD1775 in OVCAR8, suggesting that it may be gain‐of‐function of mt*TP53* rather than loss‐of‐function that mediates UPR activation.

### 2.2 AZD1775‐Triggered UPR is Induced Through NF‐*κ*B‐Dependent SASP

**Figure 2:**
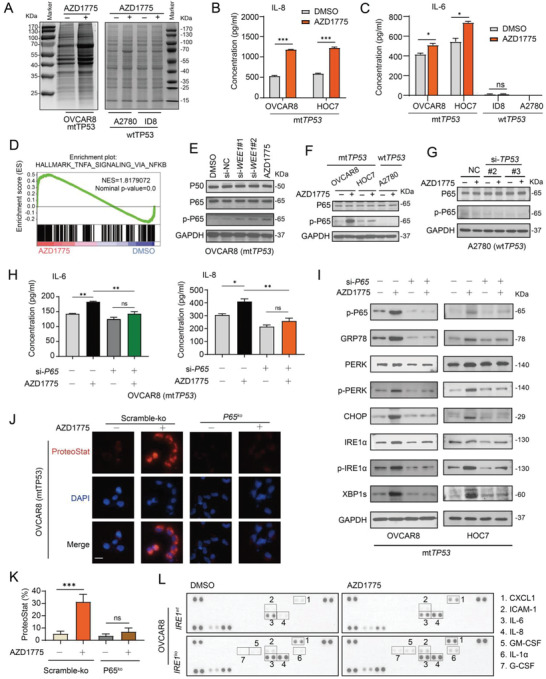
AZD1775‐triggered UPR was induced via NF‐*κ*B‐dependent SASP in *mtTP53* ovarian cancer cells. A) Intracellular soluble protein assay of OVCAR8, A2780, and ID8 cells treated with DMSO or 400 × 10^−9^ m AZD1775. B,C) Levels of two recognized SASP factors IL‐8, IL‐6 were measured by ELISA assay following 72 h treatment with DMSO or 400 × 10^−9^ m AZD1775. Error bars represent the SD of the mean (*n* = 3). *p < 0.05, ***p < 0.001, ns, not significant, as determined by the unpaired two‐tailed Student's *t*‐test. D) GSEA plot of the Hallmark pathway TNF‐*α* Signaling Via NF‐*κ*B enriched by GSEA in OVCAR8 cells treated with 400 × 10^−9^ m AZD1775 for 48 h. E) Western blot analysis of the protein expression levels of P50, P65, p‐P65 in NF‐*κ*B pathway. OVCAR8 cells were treated with DMSO or 400 × 10^−9^ m AZD1775 or transfected with scramble siRNA (si‐NC) or two *WEE1* siRNAs for 24 h. F) Western blot analysis of the protein expression levels of P65, p‐P65. *mtTP53* cells OVCAR8 and HOC7, and *TP53* wild type cells A2780 were treated with or without 400 × 10^−9^ m AZD1775 for 24 h. G) Western blot analysis of P65 and p‐P65 in A2780 cells treated with or without 400 × 10^−9^ m AZD1775 for 24 h after transfected with scramble siRNA (NC) or TP53 siRNA. #2 and #3 denote deferent *TP53* siRNAs. H) Levels of IL‐6 and IL‐8 in cell culture supernatant were measured by ELISA assay. OVCAR8 cells were transfected with or without P65 siRNA#3 (si‐P65) verified in Figure S3A (Supporting Information) and treated with or without 400 × 10^−9^ m AZD1775 for 24 h. Error bars represent the SD of the mean (*n* = 3), *p < 0.05, **p < 0.01, as determined by ANOVA with Bonferroni post hoc test. I) The level of key proteins in the UPR was determined by Western analysis. OVCAR8 and HOC7 cells were treated the same as in (H) and harvested after 72 h cultivation. J) Representative images of aggregated proteins. OVCAR8 cells with scramble‐ko or *P65*‐knockout (*p65* ^ko^) were treated with DMSO or 400 × 10^−9^ m AZD1775 for 48 h and protein aggregates were detected using the Proteostat Dye. Scale bar, 20 µm. K) The quantification of aggregated proteins shown in (J). Error bars represent the SD of the mean (*n* = 3), ***p < 0.001, ns, not significant, as determined by ANOVA with Bonferroni post hoc test. L) Expression profile of cytokines in culture supernatant was determined by chemiluminescence using a Human XL Cytokine Array (see method for details). OVCAR8 cells with scramble‐ko or *IRE1*‐knockout (*IRE1* ^ko^) were cultured in medium containing 2% serum and treated with or without 400 × 10^−9^ m AZD1775 for 48 h.

To gain insight into how AZD1775 induces UPR in mt*TP53* ovarian cancer cells, we re‐evaluated the RNA‐seq data and noted that the TNF‐*α*‐NF‐*κ*B signaling and inflammatory response pathways were significantly enriched (Figure 1A). Indeed, the expression of inflammatory cytokines, including IL‐6, IL‐8, IL‐12, CXCL5, and IFN‐*γ*, was elevated in AZD1775‐treated OVCAR8 and HOC7 cells, but remained almost unchanged in A2780 and ID8 cells (Figure S2A, Supporting Information). Interestingly, AZD1775 robustly increased the total amount of secreted proteins of OVCAR8 cells, but not in A2780 and ID8 cells (**Figure** **2**A). Concordantly, the secreted level of IL‐6 and IL‐8, two canonical cytokines of senescence‐associated secretory phenotype (SASP),^[^ ^18^ ^]^ were strongly upregulated in mt*TP53* OVCAR8 and HOC7 cells 72 h post AZD1775 treatment, but remained virtually undetectable in A2780 and ID8 cells (Figure 2B,C). This suggests that AZD1775 may burden the ER, at least in part, by inducing SASP. In line with this hypothesis, key features of senescence, including increased cell size, granularity, X‐gal‐based *β*‐galactosidase (SA‐*β*‐gal) activity, and G2M arrest were observed in OVCAR8 and HOC7 cells but not in A2780 and ID8 cells (Figure S2B–F, Supporting Information).

**Figure 3:**
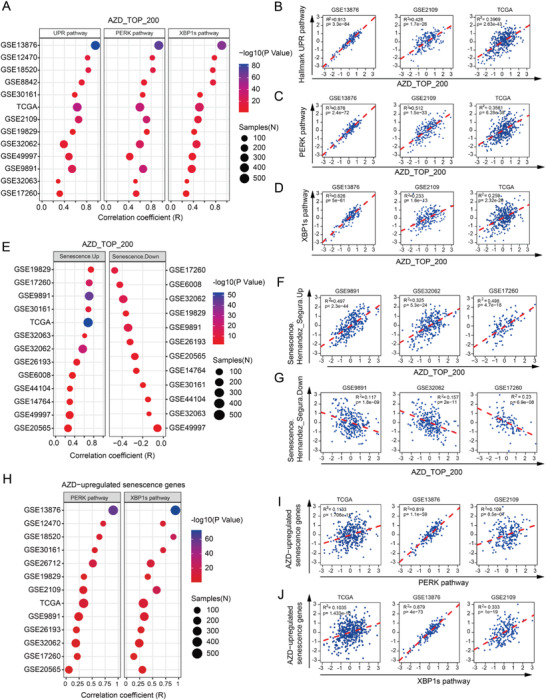
AZD1775 response, senescence, and UPR are functionally related in clinical datasets of ovarian cancer. A) the bubble chart shows the correlation coefficient between AZD1775‐induced top 200 differential genes (AZD_TOP_200) and pathways of UPR, PERK, and XBP1s in 13 different human ovarian cancer cohorts using the Pearson correlation test. B–D) Representative correlational plots of AZD_TOP_200 with Hallmark UPR pathway B), PERK pathway C), and XBP1s pathway D) in three independent ovarian cancer datasets. E) The bubble chart shows the correlation coefficient between AZD_TOP_200 and senescence signatures (Hernandes_Segura.UP (Senescence Up) and Hernandes_Segura.Down (Senescence Down)) in various ovarian cancer datasets using the Pearson correlation test. F,G) Representative correlational plots of AZD_TOP_200 with senescence up F) and senescence down G) in three independent ovarian cancer datasets. H) The bubble chart shows the correlation coefficient between AZD1775‐induced senescence genes (AZD‐induced senescence genes) and pathways of PERK and XBP1s in 13 human ovarian cancer cohorts using the Pearson correlation test. I,J) Representative correlational plots of AZD‐induced senescence genes with PERK pathway I) and XBP1s pathway J) in three independent ovarian cancer datasets.

To examine the causal relationship between SASP and UPR, we focused on the significantly enriched NF‐*κ*B pathway (Figure 2D), which is a central regulator of the proinflammatory pathway and SASP.^[^ ^19^ ^]^ WEE1 inhibition either by siRNA‐mediated knockdown or AZD1775 led to markedly upregulated level of phospho‐P65 (p‐P65) subunit of the NF‐*κ*B transcriptional complex in OVCAR8 and HOC7 cells, while p‐P65 was almost undetectable in A2780 cells (Figure 2E,F). In keeping with the effect on UPR activation, *TP53* knockdown failed to alter P65 expression in A2780 cells treated with AZD1775 (Figure 2G). NF‐*κ*B inhibition either by siRNA‐mediated *P65* knockdown (Figure S3A, Supporting Information) or CRISPR‐Cas9 mediated *P65* knockout (*P65* ^ko^) (Figure S3B, Supporting Information) alleviated AZD1775‐induced protein secretion (Figure S3C, Supporting Information), in particularly that of IL‐6 and IL‐8 in OVCAR8 and HOC7 cells (**Figure** **3**H; and Figure S3D, Supporting Information). Importantly, AZD1775‐induced activation of PERK‐CHOP and IRE1*α*‐XBP1s pathways as well as protein aggregation was significantly attenuated by *P65* knockdown (Figure 2I–K). Conversely, several SASP cytokines induced upon AZD1775 treatment was not reversed but slightly enhanced by *IRE1* knockout (*IRE1* ^ko^) (Figure 2L; and Figure S3E, Supporting Information), indicating that SASP was not downstream of IRE1*α* signaling in this context. Together, these data demonstrate that AZD1775‐triggered UPR is induced through NF‐*κ*B‐dependent SASP in mt*TP53* ovarian cancer cells.

### 2.3 AZD1775‐Induced Signature, Senescence, and UPR are Functionally Related in Clinical Datasets of Ovarian Cancer

To investigate the clinical relevance of the observed link between AZD1775 response, cellular senescence, and UPR activation, we performed bioinformatic analysis utilizing gene signatures (Table S1, Supporting Information) representing AZD1775 response, senescence, UPR, as well as PERK or XBP1s alone in multiple publicly available datasets of ovarian cancer. UPR activity was determined by the average expression of overall genes in the Hallmark UPR pathway. Gene signatures of senescence, PERK, and XBP1s were derived from previous studies by us and others.^[^ ^18^, ^20^, ^21^ ^]^ The AZD1775 signature, AZD1775_top_200, was based on the top 200 upregulated genes induced by AZD1775 in OVCAR8 RNA‐seq data. Strikingly, we observed a significant correlation between the AZD1775 signature and Hallmark UPR, PERK, and XBP1s pathways in various datasets (Figure 3A). Three examples for each pair are shown (Figure 3B–D), with the remainder shown in Figure S4A–C (Supporting Information). Furthermore, the AZD1775 signature was also strongly associated with the senescence signature (positive with senescence‐up signature and negative with senescence‐down signature) in various datasets (Figure 3E–G; and Figure S4D,E, Supporting Information).

Furthermore, we overlapped the senescence‐up signature with the OVCAR8 AZD1775 RNA‐seq data and generated an AZD1775‐induced senescence signature (Table S1, Supporting Information). This AZD1775‐induced senescence gene signature was again highly correlated with PERK and XBP1s pathway activity in a number of clinical datasets (Figure 3H–J; and Figure S4F,G, Supporting Information). Together, these results indicate that the connection between AZD1775 response, cellular senescence, and UPR activation is of significant clinical relevance.

### 2.4 AZD1775 Induces Apoptosis in TP53 Mutated Cells Through the PERK‐CHOP Branch

**Figure 4:**
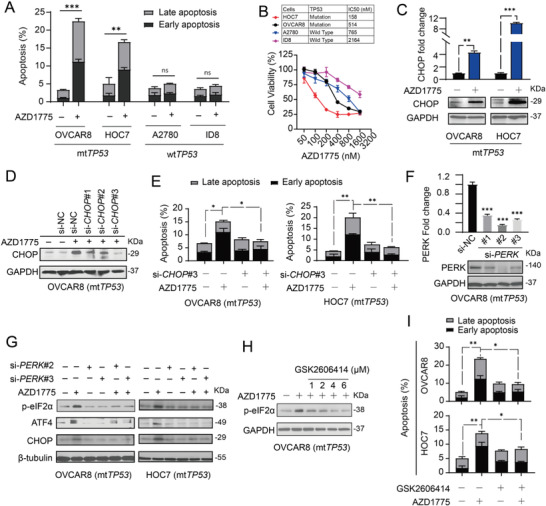
AZD1775 induced apoptosis of *TP53* mutated cells through the PERK‐CHOP branch. A) Quantification of the dead cells in ovarian cancer cell lines detected by flow cytometry. Cells were treated with or without 400 × 10^−9^ m AZD1775 for 48 h. Error bars represent the SD of the mean (*n* = 3). **p < 0.01, ***p < 0.001, ns, not significant, as determined by the unpaired two‐tailed Student's *t*‐test. B) Ovarian cancer cells were treated with a series of indicated doses of AZD1775 and each does with three biological replicates. Cell viability was measured by CCK8 after cultivation for 48 h. The half maximal inhibitory concentration (IC50) was calculated by GraphPad Prism 8.0. C) The relative mRNA and protein levels of CHOP evaluated by RT‐qPCR (up) and Western blot analysis (down). OVCAR8 and HOC7 cells were treated with 400 × 10^−9^ m AZD1775 for 48 h (RT‐qPCR) or 72 h (Western blot analysis). Data across panels represent mean ± SEM (*n* = 2). **p < 0.01, ***p < 0.001, as determined by the unpaired two‐tailed Student's *t*‐test. D) Western blot analysis of the gene silence effect by three *CHOP* siRNA after 400 × 10^−9^ m AZD1775 cultivation for 72 h. E) Quantification of dead cells in OVCAR8 and HOC7 by flow cytometry. Cells were transfected with either scrambled siRNA or CHOP siRNA#3 and incubated with or without 400 × 10^−9^ m AZD1775 for 48 h. Error bars represent the SD of the mean (*n* = 3). *p < 0.05, **p < 0.01, as determined by ANOVA with Bonferroni post hoc test. F) RT‐qPCR (up) and Western blot analysis (down) showing gene silencing by three siRNAs against PERK for 48 h in OVCAR8. The expression of scramble siRNA(si‐NC) was used as control. Data across panels represent mean ± SEM (*n* = 2). ***p < 0.001, as determined by the unpaired two‐tailed Student's *t*‐test. G) Protein levels of p‐eIF2*α*, ATF4, and CHOP in OVCAR8 and HOC7 cells after PERK silencing by two siRNAs in the absence or presence of 400 × 10^−9^ m AZD1775 for 48 h were determined by Western analysis. H) Protein expression of p‐eIF2*α* was determined by Western analysis. OVCAR8 cells were treated with indicated doses of GSK2606414 at the present of 400 × 10^−9^ m AZD1775. I) Quantification of dead cells in OVCAR8 and HOC7 by flow cytometry. Cells were pretreated with or without 1 × 10^−6^ m GSK2606414 for 24 h and then incubated with or without 400 × 10^−9^ m AZD1775 for 24 h. Error bars represent the SD of the mean (*n* = 3). *p < 0.05, **p < 0.01, as determined by ANOVA with Bonferroni post hoc test.

Next, we characterized the functional consequences of activated PERK and IRE1*α* pathways in ovarian cancer cells following WEE1 inhibition. As expected, AZD1775 significantly induced apoptosis in mt*TP53* OVCAR8 and HOC7 cells (**Figure** **4**A). In line with previous findings,^[^ ^22^ ^]^ OVCAR8 and HOC7 cells with mt*TP53* were more sensitive to AZD1775 than A2780 and ID8 cells with wt*TP53* (Figure 4B). Mirroring the enriched PERK‐mediated intrinsic apoptotic pathways in the transcriptomic analysis (Figure S1D, Supporting Information), we observed a marked increase in both mRNA and protein levels of CHOP in OVCAR8 and HOC7 cells following AZD1775 treatment (Figure 4C). *CHOP* knockdown significantly decreased AZD1775‐induced apoptosis (Figure 4D,E). Genetic inhibition of PERK reversed the activation of downstream p‐eIF2*α*, ATF4, and CHOP (Figure 4F,G), confirming that this effect was mediated by PERK rather than other eIF2*α* kinases. In keeping with this, the PERK kinase inhibitor GSK2606414 dose‐dependently blocked eIF2*α* phosphorylation in OVCAR8 cells (Figure 4H), and significantly alleviated AZD1775‐induced apoptosis (Figure 4I). These results suggest that the PERK‐eIF2*α*‐CHOP pathway plays a proapoptotic role in mt*TP53* ovarian cancer cells treated with AZD1775.

### 2.5 Inhibition of the IRE1*α*‐XBP1 Branch Promotes Apoptosis

**Figure 5:**
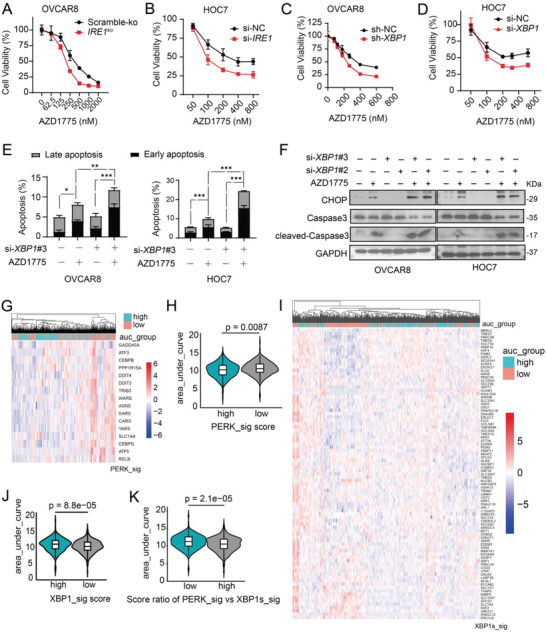
Inhibition of IRE1*α*‐XBP1 branch promoted apoptosis. A–D) Cell viability was measured by CCK8 after treated with a series of indicated doses of AZD1775 for 48 h. Biological triplicates from one representative experiment of two performed with similar results. A) OVCAR8 cells with scramble‐ko or *IRE1*‐knockout (*IRE1* ^ko^). B) HOC7 cells were transfected with scramble siRNA (si‐NC) or *IRE1* siRNA#2 verified in Figure S5A (Supporting Information). C) OVCAR8 cells transduced with either scramble (sh‐NC) or *XBP1* shRNA (sh‐*XBP1*). D) HOC7 cells were transfected with scramble siRNA (si‐NC) or XBP1 siRNA#3 verified in Figure S5B (Supporting Information). E) Quantification of dead cells in OVCAR8 and HOC7 by flow cytometry. Cells were transfected with either si‐NC or si‐*XBP1*#3 and treated with 400 × 10^−9^ m AZD1775 for 48 h. Error bars represent the SD of the mean (*n* = 3). *p < 0.05, **p < 0.01, ***p < 0.001, as determined by ANOVA with Bonferroni post hoc test. F) OVCAR8 and HOC7 cells were transfected with either si‐NC or si‐*XBP1*#2 or si‐*XBP1*#3 verified in Figure S5B (Supporting Information) followed by 400 × 10^−9^ m AZD1775 treatment for 48 h. Protein expression of CHOP cleaved and total caspase3 was determined by Western analysis. G) Heatmap of PERK signature (PERK_sig) expression of each cell from the Cancer Therapeutics Response Portal (CTRP) database grouped by area under curve (AUC) (see method for details). H) Violin plot of difference in AUC between high and low PERK_sig scores as shown in G). *P* value was determined using Wilcoxon test. I) Heatmap of XBP1s signature (XBP1s_sig) scores of each cell from the CTRP database grouped by AUC. J) Violin plot of difference in AUC between high and low XBP1s_sig scores as shown in I). *P* value was determined using Wilcoxon test. K) Violin plot of difference in AUC between high and low score ratio of PERK_sig versus XBP1s_sig. *P* value was determined using Wilcoxon test.

In terms of the function of IRE1*α*‐XBP1s pathway, cell viability assay showed increased sensitivity of OVCAR8 and HOC7 cells to AZD1775 when IRE1*α* or XBP1 was genetically depleted (**Figure** **5**A–D; and Figure S5A–C, Supporting Information). Furthermore, RNA‐seq data and flow cytometry revealed that the AZD1775‐induced apoptotic effect was augmented after XBP1 depletion (Figure 5E; and Figure S5D, Supporting Information), where concomitant increased expression of AZD1775‐induced CHOP and cleaved‐caspase 3 was observed (Figure 5F). These results suggest that inhibition of the IRE1*α*‐XBP1s branch may promote apoptosis.

To further test this, we utilized the Cancer Therapeutics Response Portal (CTRP) database^[^ ^23^ ^]^ to analyze the correlation of sensitivity to WEE1 inhibitors with PERK and XBP1s activity, represented by the above described gene signatures of PERK and XBP1s (Table S1, Supporting Information). Strikingly, higher PERK activity was significantly associated with sensitivity to WEE1 inhibition (Figure 5G,H), while higher XBP1s activity was tightly linked to resistance to WEE1 inhibitors (Figure 5I,J). In addition, cells with a higher ratio of PERK/XBPB1s were more sensitive to WEE1 inhibitors (Figure 5K). Together, these data implicate the divergent roles of PERK and IRE1*α* pathways in response to AZD1775 in mt*TP53* ovarian cancer cells.

### 2.6 AZD1775 and MKC8866 are Synergistic in mt*TP53* Ovarian Cancer Cells

**Figure 6:**
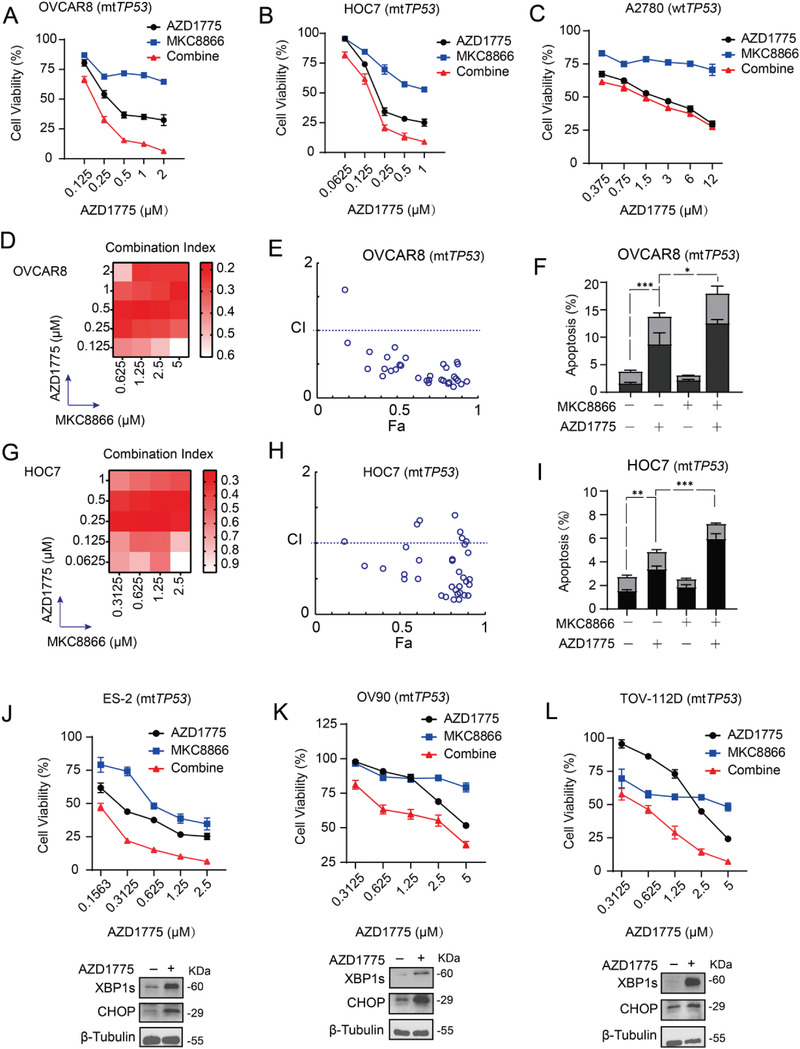
AZD1775 and MKC8866 were synergistic in *mtTP53* ovarian cancer cells. A–C) Cell viability of OVCAR8, HOC7, and A2780 was measured by CCK8 after treated with a series of indicated doses of AZD1775 and MKC8866 at specified ratios of 1:2.5 for 48 h. Biological triplicates from one representative experiment of two performed with similar results. D,G) Heatmap of combination index (CI) values for combination treatment between AZD1775 and MKC8866. E,H) CI values for the entire fraction affected (Fa) of OVCAR8 and HOC7. F,I) Quantification of dead cells in OVCAR8 and HOC7 by flow cytometry. Cells were treated with or without 10 × 10^−6^ m MKC8866 and incubated with or without 400 × 10^−9^ m AZD1775 for 48 h. Error bars represent the SD of the mean (*n* = 3). *p < 0.05, **p < 0.01, ***p < 0.001, as determined by ANOVA with Bonferroni post hoc test. J–L) Three TP53‐mutant cell lines (ES‐2, OV90, and TOV‐112D) were treated with indicated doses of AZD1775 and MKC8866 at specified ratios of 1:2. Cell viability (up) was measured by CCK8 assay after 48 h cultivation. Biological triplicates from one representative experiment of two performed with similar results. Protein expression of XBP1s and CHOP was determined by Western analysis (down) after cells treated with or without 400 × 10^−9^ m AZD1775 for 72 h.

Based on these results, we then evaluated whether pharmacological inhibition of IRE1*α*‐XBP1s axis would augment the antitumor effect of AZD1775 on mt*TP53* ovarian cancer cells. To this end, we combined AZD1775 with MKC8866, a specific IRE1*α* RNase inhibitor previously described in multiple cancer models.^[^ ^10^, ^15^, ^24^ ^]^ We first verified that MKC8866 robustly suppressed *XBP1* splicing in OVCAR8 cells (Figure S6A, Supporting Information). Next, we treated the cells with a fixed ratio of AZD1775 and MKC8866 (1:2.5) at a series of concentrations. In OVCAR8 and HOC7 cells, coadministration of AZD1775 and MKC8866 showed more potent inhibition of cell viability than either drug alone (**Figure** **6**A,**6**B), a phenotype not occurred in A2780 and ID8 cells (Figure 6C; and Figure S6B, Supporting Information). To further explore the synergistic effect of AZD1775 and MKC8866, we designed a matrix of concentrations, wherein multiple combinatorial ratios reached significant synergy in both OVCAR8 and HOC7 cells characterized by the combination index (CI) < 1 (Figure 6D,E,G,H). Flow cytometry confirmed that MKC8866 significantly enhanced AZD1775‐induced apoptosis in OVCAR8 and HOC7 cells (Figure 6F,I).

To evaluate the generalizability of synergy between AZD1775 and MKC8866, we assessed three additional mt*TP53* human ovarian cancer cell lines, ES‐2, OV90, and TOV‐112D. XBP1s and CHOP levels were markedly induced after AZD1775 exposure in these cells (Figure 6J–L). Coadministration of AZD1775 and MKC8866 similarly suppressed cell viability in a synergistic manner (Figure 6J–L). Together, these results demonstrate that AZD1775 and MKC8866 combination leads to synergistic inhibition on the viability of mt*TP53* ovarian cancer cells.

### 2.7 AZD1775 Synergizes with MKC8866 in PDX Models of mt*TP53* Ovarian Cancer

**Figure 7:**
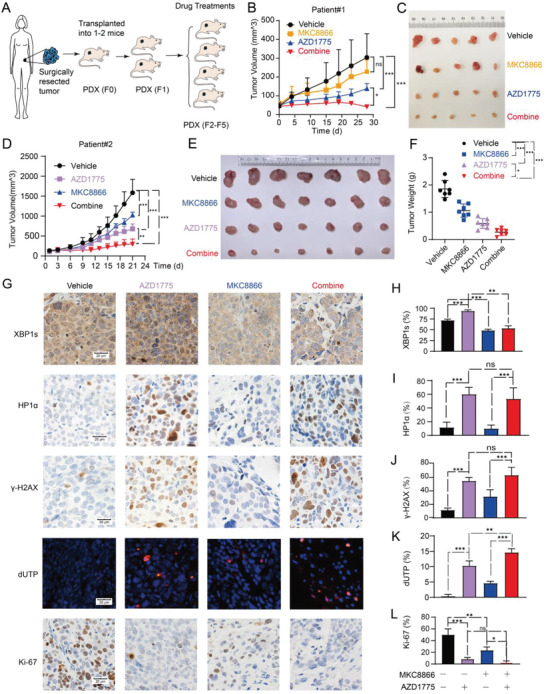
AZD1775 synergizes with MKC8866 in *MtTP53* PDXs of HGSOC. A) Schematic diagram of the generation of PDX models. The patient‐derived tumor materials were xenografted and passaged in nude mice. B) Nude mice bearing xenografted tumors of patient#1 were randomly divided into four groups and treated with either vehicle or 60 mg kg^−1^ AZD1775 (5 days on and 2 days off) or 300 mg kg^−1^ MKC8866 daily or combined treatment of the two drugs. Tumor growth was recorded every 4 days. Error bars represent the SD of the mean (*n* = 5 each group). *p < 0.05, ***p < 0.001, ns, not significant, as determined by ANOVA with Bonferroni post hoc test. C) Image of the tumor specimens of patient#1 after harvested on the 28th day of drugs administration. D–F) Nude mice bearing xenograft tumors of patient#2 were randomly divided into four groups (*n* = 7 each group). Nude mice were treated in the same way as described in B). D) Tumor growth of patient#2 was recorded every other day until day 21. Error bars represent the SD of the mean (*n* = 7). **p < 0.01, ***p < 0.001, as determined by ANOVA with Bonferroni post hoc test. E) Image of the tumor specimens of patient#2 after harvested. F) Tumor weight of patient#2 was measured after harvested. Error bars represent the SD of the mean (*n* = 7). *p < 0.05, ***p < 0.001, as determined by ANOVA with Bonferroni post hoc test. G) Representative images of immunohistochemical staining and dUTP labeling. Scale bar, 20 µm. H–L) quantification of XBP1s, HP1*α*, *γ*‐H2AX, apoptosis (dUTP), and Ki‐67 foci in harvested tumors as shown in G). Error bars represent the standard deviation of the mean (*n* = 5). *p < 0.05, **p < 0.01, ***p < 0.001, ns, not significant, as determined by ANOVA with Bonferroni post hoc test.

We further investigated the combinatorial efficacy of AZD1775 and MKC8866 in HGSOC PDX models with mt*TP53*. The PDX models were established and passaged as schematically depicted in **Figure** **7**A. In the PDX model of patient#1, AZD1775 monotherapy showed significant antitumor activity, while MKC8866 modestly repressed tumor growth. Coadministration of AZD1775 and MKC8866 significantly induced tumor regression over the 30‐day treatment period (Figure 7B,C). For PDX of patient#2, both AZD1775 and MKC8866 monotherapy significantly attenuated tumor growth, while coadministration still reached significant synergy (Figure 7D–F). Immunohistochemistry confirmed that AZD1775 increased XBP1s, HP1*α* (a marker of cellular senescence), and *γ*‐H2AX (Figure 7G–J), consistent with the observations in vitro. Combination treatment significantly augmented AZD1775‐induced apoptosis characterized by increased terminal deoxynucleotidyl transferase‐mediated dUTP‐biotin nick end labeling assay (TUNEL) signals (Figure 7G,K), and in the meantime reduced proliferation as represented by Ki67 staining (Figure 7G,L). Together, these results indicate that AZD1775 synergizes with MKC8866 in mt*TP53* PDX models of ovarian cancer.

## 3 Discussion

**Figure 8:**
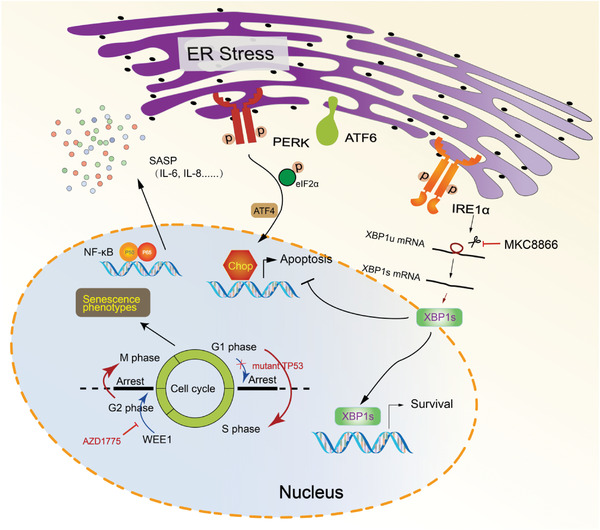
Schematic diagram of the correlation between WEE1 inhibition and ER stress. Restriction of the G2/M checkpoint by WEE1 inhibitor AZD1775 results in disturbed proteostasis and UPR activation in ovarian cancer cells with *TP53* mutations, which is mediated by the NF‐*κ*B‐governed SASP. AZD1775 activates the PERK and IRE1*α* branches of UPR in ovarian cancer cells exclusively with mt*TP53*. PERK facilitates apoptotic signaling via activating CHOP upon AZD1775 treatment, whereas IRE1*α*‐XBP1s promotes survival, which confers the synergistic antitumor effect of AZD1775 and MKC8866.

The bilateral regulatory interplay between cell cycle regulation and proteostasis is currently an exciting area of research in cancer biology. Our results showed that restriction of the G2/M checkpoint by WEE1 inhibition resulted in disturbed proteostasis and UPR activation in ovarian cancer cells with *TP53* mutations, which was mediated by the NF‐*κ*B‐governed SASP. WEE1 inhibitor AZD1775 activated the PERK and IRE1*α* branches of UPR that exert distinct effect on cell survival. This provided a therapeutic opportunity for synergistic antitumor effect between AZD1775 and MKC8866, which was observed in various ovarian cancer cell lines and PDX models (**Figure** **8**).

*TP53* mutations are essentially universal in HGSOC.^[^ ^25^ ^]^ In addition to loss of the tumor suppressor function, p53 mutations often lead to acquired oncogenic functions, a phenomenon known as mutant p53 gain of function (GOF).^[^ ^26^ ^]^ The evidence obtained from the models tested in this study implicated that AZD1775‐induced senescence and NF‐*κ*B mediated SASP were dependent on the mt*TP53* GOF activity, which is consistent with several previous reports showing that the mutant p53 GOF is indispensable for prolonged NF‐*κ*B activation and chronic inflammation.^[^ ^27^, ^28^, ^29^ ^]^ It is speculated that the potential interaction between mutant p53 and NF‐κB can lead to prolonged activation of NF‐κB and elevation of downstream proinflammatory cytokines through its long‐term stability at κB sites.^[^ ^30^ ^]^ Considering the highly diverse alterations of p53 will lead to different GOF consequences, the mechanisms how and to what extent the different mutations affected the AZD1775 induced UPR activation will need further investigation.

To date, most studies have investigated the roles of IRE1*α* and PERK as transcriptional regulators of DDR proteins.^[^ ^31^ ^]^ In contrast, much less is known as to whether DDR factors participate in the regulation of UPR signaling. Our results showed that inhibition of the DNA‐damage response (DDR) checkpoint resulted in ER stress and UPR activation via inducing senescence and SASP, thereby providing an indirect regulatory mechanism in this regard. PERK arm appeared to contribute to the cytotoxic effect of AZD1775 via CHOP, whereas IRE1*α*‐XBP1s pathway seemed cytoprotective and possibly confer resistance to the WEE1 inhibitor. This is in line with the established role of this branch in many other tumors where it functions as a driver of drug resistance and metastasis.^[^ ^32^, ^33^, ^34^ ^]^ Targeting this pathway either alone or in combination with other drugs (such as chemotherapeutic agents) have demonstrated exciting efficacy in preclinical models of prostate cancer, breast cancer, and multiple other malignancies.^[^ ^10^, ^15^, ^24^ ^]^

Recent studies have demonstrated that AZD1775 exhibits single‐agent activity in both wild‐type or mutant *TP53* cell line subsets,^[^ ^35^, ^36^ ^]^ yet better patient stratification to improve the selectivity of this treatment is still needed. Our extensive bioinformatics analyses using various clinical cohorts of advanced ovarian cancer patients confirmed a striking correlation between AZD1775 responsiveness, UPR activation, and senescence. These findings suggest that UPR activation and senescence status may offer a new perspective for predicting the responsiveness of *TP53* mutated ovarian tumors to WEE1 inhibitors.

In conclusion, our study provides new evidence on the interaction between genomic instability and proteostatic stress in cancer cells. PERK and IRE1*α* arms of the UPR are strongly induced and elicit distinct roles in mediating the response of p53 mutant ovarian cancer cells to AZD1775. The remarkable synergy between coinhibition of WEE1 and IRE1*α* in various model systems potentiate the clinical evaluation in patients with advanced ovarian cancer.

## 4 Experimental Section

### Cell Lines and Cell Culture

The ovarian cancer cell lines OVCAR8 and HOC7 were obtained from M.D. Anderson Cancer Center characterized Cell line Core. A2780 was obtained from Applied Biological Materials Inc. (ABM) Canada, while ES‐2, TOV‐112D, and OV90 were obtained from the American Type Culture Collection (ATCC). The murine ovarian cancer cell line ID8 was a gift from K. Roby (University of Kansas, Lawrence, KS), derived from spontaneous malignant transformation of C57BL/6 mouse ovarian surface epithelium cells.^[^ ^37^ ^]^ HOC7, OVCAR8, and A2780 were cultured in RPMI 1640 Medium (CAT#72 400 047, Thermo Fisher Scientific) supplemented with 10% fetal bovine serum. ES‐2 cells were cultured in McCoy's 5A medium containing 10% fetal bovine serum. The base medium for OV90 and TOV‐112D cell line is a 1:1 mixture of MCDB 105 medium containing a final concentration of 1.5 g L^−1^ sodium bicarbonate and medium 199 containing a final concentration of 2.2 g^−1^ L^−1^ sodium bicarbonate supplemented with 15% fetal bovine serum. ID8 cells were cultured in Dulbecco's modification of Eagle's medium (DMEM) containing 10% fetal bovine serum. All medium was supplemented with penicillin and streptomycin. All cell lines were fingerprinted by short tandem repeat assays and verified to be free of mycoplasma contamination before use and incubated at 37 °C in an incubator with 5% CO_2_.

### Chemical Compounds

AZD1775 (CAT#S1525) and GSK2606414 (CAT#S7307) were purchased from Selleck. MKC8866 was obtained from Fosun Orinove PharmaTech, Inc. All these chemical compounds were dissolved in Dimethylsulfoxide (DMSO) before being used in vitro experiments.

### Cell Viability Assay

3000–5000 cells were seeded in 96‐well plates 24 h prior to indicated drug administration. All experiments were performed in triplicates. After 48 h drug treatment, cell viability was assessed with a cell counting kit‐8 (CCK‐8, Dojindo Laboratories, Japan) according to manufacturer instructions. The absorbance of optical density (OD) values was measured at 450 nm using a microplate reader (Bio‐Rad). The relative cell viability was calculated by setting the control group as a reference. The graphics were generated in GraphPad prism 8.0.

### Flow Cytometry Analysis

Cancer cells were seeded in 6‐well plates and treated for 48 h before preparing for flow cytometry. For apoptosis analysis, harvested cells were resuspended in 200 µL phosphate‐buffered saline (PBS) and incubated with Annexin V and propidium iodide for 10 min at room temperature in the dark using a FITC Annexin V Apoptosis Detection Kit I (CAT#556 547, BD Biosciences). For cell cycle analysis, harvested cells were fixed using 75% ice‐cold ethanol in −20 ℃ overnight followed by 0.25% Triton 100 to permeabilize the cell membrane. Cells were incubated with 300 µL RI/RNase staining buffer (CAT#550 825, BD Pharmingen) for 10 min at room temperature in the dark before detection. All samples were assessed on a Beckman Coulter flow cytometer. At least 10 000 events were assessed per sample. FlowJo‐V10 software was used to quantify cell populations.

### Proteostat Aggresome Detection

Cells were seed on the glass slides and treated with 400 × 10^−9^ m AZD1775 for 72 h. Proteostat Aggresome was detected following the guide of PROTEOSTAT Aggresome Detection Kit (Cat#ENZ‐51035, BD Biosciences). Briefly, cells were firmed with 4% formaldehyde for 30 min at room temperature and then washed three times with PBS. slides were transferred into a permeabilizing solution (0.5% Triton X‐100, 3 × 10^−3^ m ethylene diamine tetraacetie acid (EDTA), pH 8) on ice for 30 min. After washing with PBS buffer, slides were incubated with Proteostat dye at 1:5000 dilution for 30 min in the dark followed by DAPI (4′,6‐diamidino‐2‐phenylindole) staining to indicate nuclei. Cells were observed under a microscope and photographed.

### Western Blot Analysis

Western blot was performed as previously described.^[^ ^37^ ^]^ Harvested cells were lysed, sonicated, centrifuged, and the supernatants were collected. Protein concentration was measured using Coomassie (CAT#ST1119, CAT#P0006C, Beyotime). 20 µg of total protein was used for all blots. Sodium dodecyl sulfate – polyacrylamide gel electrophoresis (SDS‐PAGE) separations were performed, and the samples were transferred to polyvinylidene fluoride (PVDF) membranes. Gels were blocked with 5% bovine serum albumin (BSA) before incubation with primary antibodies at 4 °C overnight. 1:5000 secondary antibody (Abclonal) was added for 1 h at room temperature on the next day. Bands were visualized by WesternBright ECL using a Western blotting detection kit (CAT#K‐12045‐D50, Advansta) in the ChemiDoc Imaging System (Bio‐Rad). The following antibodies were used: Anti‐WEE1 (1:1000, Abcam#ab233540), Phospho‐CDK1 (Tyr15) (1:500, ABclonal#AP0016), Anti‐*γ*H2AX (phospho‐S140) (1:1000, Abcam#ab‐22551), GRP78 (1:1000, CST#3177), PERK (1:1000, CST#3192), phospho‐PERK (1:1000, Abcam#ab192591), ATF6 (1:1000, CST#65 880), IRE1*α* (1:1000, CST#3294), phospho‐IRE1*α* (Ser724) (1:1000, Thermo Fisher Scientific#PA1‐169), XBP1s (1:1000, CST#12 782), CHOP (1:500, Proteintech#15204‐1‐AP), p53 (1:1000, Proteintech#10442‐1‐AP), Phospho‐eIF2*α* (1:1000, CST#9721), *β*‐Tubulin (1:1000, ABclonal#AC015), Caspase‐3 (1:1000, CST#9662), Cleaved‐Caspase‐3 (1:1000, CST#9661), NF‐*κ*B P65 (1:1000, CST#8242S), Phospho‐NF‐*κ*B P65 (1:1000, CST#3033), NF‐*κ*B p50 (1:1000, CST#13 586), GAPDH (1:5000, ABclonal#A19056), HRP Goat Anti‐Rabbit IgG (H+L) (≈1:5000–10 000, ABclonal#AS014), HRP Goat Anti‐Mouse IgG (H+L) (≈1:5000–10 000, ABclonal#AS003).

### Senescence *β*‐Galactosidase (SA‐*β*‐gal) Staining

Cells were seeded in 6‐well plates and treated for 48 h before detection. SA‐*β*‐gal staining was conducted using the Senescence *β*‐Galactosidase Staining Kit (CAT#C0602, Beyotine) according to manufacturer instructions. The cells were incubated with SA‐*β*‐gal staining buffer at 37 °C in an incubator without CO_2_ for 12 h and washed twice using PBS. The ratio of senescence cells was conducted according to the proportion of stained blue cells compared to total cells.

### RNA Extraction and RT‐Qpcr

Total RNA was isolated from cultured cells using a total RNA extraction kit (CAT#DP419, TIANGEN). cDNA was synthesized using RevertAid First Strand cDNA Synthesis Kit (CAT#K1622, Thermo Fisher Scientific) on an RT‐PCR System (Bio‐Rad). RT‐qPCR was performed on a CFX Connect quantitative real‐time PCR System (Bio‐Rad) using SYBR Green Master Mix (Vazyme; cat#R223‐01). Relative mRNA expression was determined by the ΔΔCt method and normalized by *β*‐ACTIN. The sequences of primers used are listed as following

**Table:** 

Human genes	Forward primer 5“‐3”	Reverse primer 5“‐3”
ACTIN	CATGTACGTTGCTATCCAGGC	CTCCTTAATGTCACGCACGAT
IL‐6	ACTCACCTCTTCAGAACGAATTG	CCATCTTTGGAAGGTTCAGGTTG
IL‐8	CCATCTTTGGAAGGTTCAGGTTG	AACCCTCTGCACCCAGTTTTC
CXCL5	AGCTGCGTTGCGTTTGTTTAC	TGGCGAACACTTGCAGATTAC
IFN *γ*	TCGGTAACTGACTTGAATGTCCA	TCGCTTCCCTGTTTTAGCTGC
IL‐12	CCTTGCACTTCTGAAGAGATTGA	ACAGGGCCATCATAAAAGAGGT
CHOP	GGAAACAGAGTGGTCATTCCC	CTGCTTGAGCCGTTCATTCTC
XBP1s	AGTCCGCAGCAGGTGCAG	CTTCCAGCTTGGCTGATGAC

### Enzyme‐Linked Immunosorbent Assay (ELISA)

OVCAR8, HOC7, ID8, and A2780 cells were treated for 48 h in complete media and then incubated for another 24 h in medium without serum before collecting supernatants. Cell debris was removed by centrifugation. Human IL‐6 and IL‐8 were detected by QuantiCyto Human ELISA kit (High Sensitivity) (IL‐6 CAT#EHC007(H).96, IL‐8 CAT# EHC008(H).96, Neobioscience) according to the manufacturer instructions. OD values were measured at 450 nm using a microplate reader (Bio‐Rad). The concentrations of the samples were obtained according to a standard curve.

### Intracellular Soluble Proteins Assay

The cells after different treatments were collected and washed with PBS. 1 × 10^5^ cells were taken into a 1.5 mL EP tube and resuspended with 100 µL PBS. The cells were frozen and thaw twice using liquid nitrogen and a heating block set at 25 °C. Briefly vortex the tubes, and the cellular debris were pelleted by centrifuging the tubes containing the cell lysates at 20 000 g for 20 min at 4 °C. An aliquot of 30 µL from each cleared cell line lysate was mixed with the SDS‐PAGE loading buffer and loaded on separate lanes in SDS‐PAGE gel for separations. The gel was stained using Coomassie Brilliant Blue Stain Kit (CAT#G2012, Servicebio) according to the manufacturer instructions. After detained, photographs were taken using the ChemiDoc Imaging System (Bio‐Rad).

### Cytokine Array

Cells were treated for 72 h in a complete medium, and the supernatants were collected for detection. Cytokines were detected by Proteome Profiler Human Cytokine Array (Cat#ARY005B, BD Biosciences) according to the instructions of the manual. Briefly, the array membrane was incubated with supernatant at room temperature for 1 h. After being washed three times, the array was treated with streptavidin HRP for 30 min at room temperature on a rocking platform shaker. Arrays were visualized by WesternBright ECL using a Western blotting detection kit (CAT#K‐12045‐D50, Advansta) in the ChemiDoc Imaging System (Bio‐Rad). Mean spot pixel density was quantified using Image Lab software 6.0.1.

### Electron Microscopy

OVCAR8, HOC7, ID8, and A2780 cells were seeded in 10 cm dishes and treated with AZD1775 or DMSO for 48 h. The medium was discarded, and the adherent cells were fixed using an electron microscope fixed solution (CAT#G1102, Servicebio). Cells were then postfixed with 1% osmium tetroxide, dehydrated by sequential extraction with graded ethanol concentrations from 50% to 100%, and embedded in Embed 812 resin. Thin sections of 60–80 nm were cut on an ultramicrotome (Leica UC7, Leica), stained with saturated uranyl acetate and Reynolds lead citrate, and examined at room temperature using a transmission electron microscope (Tecnai G^2^ 20 TWIN; FEI). Images were captured and processed using Adobe Photoshop software.

### RNA Interference

Cells were seeded into 6‐well plates at ≈30–40% confluence, and RNA interference (RNAi) transfections were performed using Lipofectamine 3000 Transfection Reagent (Invitrogen, USA) according to the manufacturer's instructions. siRNAs were transfected at 100 × 10^−9^ m final concentration, and the medium was replaced after transfection for 12–18 h. The cells were cultured for 24–48 h before being used for other tests. Western blot analyses or RT‐qPCR were used to identify gene knockdown. All siRNAs used in this study were purchased from Sigma and listed as following

**Table:** 

siRNA	#1	#2	#3
Human TP53	SASI_Hs02_00302766	SASI_Hs02_00302767	SASI_Hs02_00302768
Human WEE1	SASI_Hs01_002 26779	SASI_Hs02_00335570	
Human P65	SASI_Hs01_00171090	SASI_Hs01_00171091	SASI_Hs01_00171092
Human CHOP	SASI_Hs01_00153013	SASI_Hs02_00336880	SASI_Hs01_00153015
Human PERK	SASI_Hs01_00096844	SASI_Hs01_00096845	SASI_Hs01_00096846
Human IRE1	SASI_Hs01_00194923	SASI_Hs01_00194924	SASI_Hs02_00331841
Human XBP1	SASI_Hs02_00313590	SASI_Hs02_00313591	SASI_Hs02_00313592

### shRNA Viral Infection

The HBLV‐h‐XBP1 shRNA1‐ZsGreen‐PURO Leni‐virus (sh‐XBP1) and HBLV‐ZsGreen‐PURO NC control Leni‐virus (sh‐Ctrl) were purchased from Hanbio (Shanghai, China). For lentiviral transduction, OVCAR8 cells were seeded in 6‐well plates at ≈30–40% confluence. The lentivirus was added at a multiplicity of infection (MOI) of 5. Cells were exposed to lentivirus for 48 h and selected with 2 µg mL^−1^ puromycin for 7 days. Western blot analyses were used to identify gene knockdown.

### CRISPR‐Cas9 Mediated Gene Editing in cells

Human *P65 TP53*, *IRE1* sgRNAs sequence were obtained from GenScript (https://www.genscript.com/). The sgRNAs sequence used are listed as following

**Table:** 

sgRNA	Forward primer	Reverse primer
Human‐P65 #1	CACCGAGGGACAGTGCGCATCTCCC	AAACGGGAGATGCGCACTGTCCCTC
Human‐P65 #2	CACCGCGCTTCCGCTACAAGTGCG	AAACCGCACTTGTAGCGGAAGCGCC
Human‐P65 #3	CACCGGACAGATCAATGGCTACAC	AAACGTGTAGCCATTGATCTGTCCC
Human‐IRE1 #1	CACCGCTTGTTGTTTGTGTCAACGC	AAACGCGTTGACACAAACAACAAGC
Human‐IRE1 #2	CACCGGCCTCGGGGTGAGTGACCG	AAACCGGTCACTCACCCCGAGGCCC
Human‐IRE1 #3	CACCGAGTCCTCGCCATGCCGGCC	AAACGGCCGGCATGGCGAGGACTCC
Human‐TP53 #1	CACCGCATGGGCGGCATGAACCGG	AAACCCGGTTCATGCCGCCCATGC
Human‐TP53 #2	CACCGTGAGCGCTGCTCAGATAGCG	AAACCGCTATCTGAGCAGCGCTCAC
Human‐TP53 #3	CACCGCCCCGGACGATATTGAACAA	AAACTTGTTCAATATCGTCCGGGGC

All sgRNAs were synthesized by TsingKe Co., Ltd. sgRNAs were cloned into the pLentiCRISPR v2 vector by restriction endonucleases BsmBI‐v2(CAT#0739S, NEBiolabs) and T4 DNA Ligase (CAT#2011A, Takara). The ligation products were transferred into DH‐5*α* competent, and all constructs were confirmed by DNA sequencing.

To create stable cell lines, the pCMV‐dR8.91 lentiviral packaging plasmid, pCMV‐VSVG envelope plasmid and the pLentiCRISPR v2 vector, sgRNA‐pLentiCRISPR v2 constructs were used to cotransfect HEK293T cells. HEK293T cells were seeded into 10 cm petri dishes to 80% confluence and transfections were performed using Lipofectamine 3000 Transfection Reagent (Invitrogen, USA). For each dish, transfection was performed using 18.75 µL of Lipofectamine 3000, 25 µL of P3000 Reagent, 4.2 µg of mutation constructs, 4.2 µg of pCMV‐dR8.91 plasmid, and 4.2 µg of pCMV‐VSVG plasmid. After 15 min of incubation at room temperature, the mixture was added to the HEK293T cells medium. Virus supernatant was collected 48 h post‐transfection and filtered through a 0.45 µm polyethersulfone filter. OVCAR8, HOC7, A2780, and ID8 cells growing on six‐well plates to ≈30–40% confluence was treated with 2 mL of the obtained virus suspension, 1 mL complete medium supplemented with 10 µg mL^−1^ polybrene (YEASEN, CAT#40804ES76). 2 µg mL^−1^ puromycin screening was conducted 48 h postinfection. After about 4 weeks of selection, stable clones were picked. Western blot analyses and DNA sequencing were used to identify gene knockout.

### Immunohistochemical (IHC) Staining

The procedure is as described previously.^[^ ^37^ ^]^ In brief, tissues were fixed overnight in 4% paraformaldehyde and embedded in paraffin. All immunohistochemical staining was performed on 4 µm sections. After deparaffinization, rehydration, antigen unmasking, and endogenous peroxidase blocking, sections were blocked in 5% bovine serum albumin (BSA) with 0.1% Triton X‐100 in PBS. Sections were incubated overnight at 4 °C with primary antibodies (Ki‐67 antibody (CAT#9129, CST, 1:400); XBP1s antibody (CAT#647 502, BioLegend, 1:100); HP1*α* antibody (CAT#2616, CST, 1:200); *γ*‐H2AX antibody (CAT#9718, CST, 1:200). Tissue sections were then stained using an HRP‐DAB Immunohistochemistry kit (CAT#G1212, Servicebio). Staining scores were assigned using a semiquantitative five‐category grading system as previously described.^[^ ^38^ ^]^

### dUTP Detection

Tissues were fixed overnight in 4% paraformaldehyde and embedded in paraffin. The experiment was performed on 4 µm sections. Apoptotic cells were detected by TUNEL labeling with fluorescein‐dUTP using One Step TUNEL Apoptosis Assay Kit (CAT#1089, Beyotime). After deparaffinization, rehydration, the sections were incubated with protein K, TUNEL mixture, and DAPI. Sections were washed using PBS and observed under a microscope and photographed. The ratio of positive cells assigned the staining scores.

### RNA Sequencing and Bioinformatic Analysis

Purified total RNA from cells was extracted using RNA extraction kit (TIANGEN, CAT#DP419) according to the manufacturer's instructions. The Beijing Genomics Institute (BGI, Shenzhen, China) conducted RNA sequencing and performed on the BGISEQ‐500 platform. Gene expression and changes were aligned to hg38 or mm10 using STAR.^[^ ^39^ ^]^ The relative abundance of mRNAs was normalized and presented as fragments per kilobase of transcript per million mapped reads (FPKM). Differential expression analysis was conducted using R statistical software (x64, version 4.0.2) in conjunction with the DESeq2 package.^[^ ^40^ ^]^ All sequence data sets had been submitted to GEO (GSE166417). The Gene Ontology (GO) of differentially expressed genes (DEGs) was performed by the cluster Profiler R package.^[^ ^41^ ^]^ The cut‐off criteria for DEGs were |logFC| ≥ 1 and false discovery rate (FDR) < 0.05. Gene set enrichment analysis (GSEA) was performed using the software provided by the Broad Institute.^[^ ^42^, ^43^ ^]^ The algorithm of random sampling was 1000 permutations. Significant gene sets were determined by FDR < 0.05 and normalized enrichment score (NES) >1. Human ovarian cancer datasets were obtained from the GEO database (https://www.ncbi.nlm.nih.gov/geo/) and the Cancer Genome Atlas (TCGA) database. Spearman correlation was evaluated using published ovarian cancer expression datasets, including GSE13876, GSE12470, GSE18520, GSE8842, GSE2109, GSE32062, GSE26712, GSE19829, GSE17260, GSE9891, GSE30161, GSE32063, GSE26193, GSE6008, GSE44104, GSE14764, GSE49997, GSE20565, and TCGA.

### Correlation Analysis of AZD1775 Sensitivity and PERK_sig or XBP1s_sig Pathway in the Cancer Cell Line

The analysis was conducted as previously described.^[^ ^37^ ^]^ In brief, the gene expression data of all cell lines were obtained from the Cancer Cell Line Encyclopedia (CCLE) database (https://portals.broadinstitute.org/ccle), and the expression of PERK_sig or XBP1s_sig was scored by the mean expression value of all genes in the pathway in each cell line. The cell lines were then divided into different groups according to the scores. In the meantime, based on the drug area under the curve (AUC) values of AZD1775 from CTRP (https://portals.broadinstitute.org/ctrp/), the sensitivity data of AZD1775 were obtained. Cell lines were divided into three equal parts according to AUC values. The low AUC part was defined as the AZD1775‐sensitive group, while the high part as the AZD1775‐resistant group. A heatmap for PERK_sig or XBP1s_sig pathway genes in the AZD1775‐resistant and ‐sensitive groups was generated using pheatmap. Wilcoxon test was used for significant difference tests.

### Patient‐Derived Xenografts (PDX) Models

All animal experiments were approved by the Animal Experiment Ethics and Medical ethics Committee of Tongji Hospital (Permit Number: TJH‐201909004). The permission of using tissue samples from human were obtained by the Medical Ethics Committee of Tongji Medical College (Permit Number: S080). Female nude mice (6–8 weeks old) were purchased from Beijing Vital River Laboratory Animal Technology Co. Ltd and housed under sterile conditions at the Laboratory Animal Care Center of Tongji Hospital. All animal experiments were conducted in compliance with the National Institute of Health guidelines for animal research. PDXs were established by subcutaneous transplantation of tumor fragments (3 × 3 × 3 mm^3^) into the flanks of nude mice ≈8–10 weeks of age (F0). PDXs were passaged at least twice but no more than five times (F2‐5) before drug administration. For treatments, mice were randomly divided into 4 groups (*n* = 5–10 per group) with tumor volumes of 100–150 mm^3^. Each group was treated with either vehicle, AZD1775 (60 mg kg^−1^, 5 days on and 2 days off), or MKC8866 (150 mg kg^−1^ d^−1^, every day), or a combination of AZD1775 and MKC8866 treatment via oral gavage. AZD1775 was dissolved in 2% DMSO + 30% PEG 300 + 5% Tween 80 + 63% ddH_2_O. MKC8866 was formulated in 1% microcrystalline cellulose in simple syrup (50%). Tumor volumes were measured every 3 days and calculated using the V = (L × W^2^)/2 (L: length; W: width). Mice were treated for 3–4 weeks and sacrificed for tissue analysis.

### Drug Combination Analysis

OVCAR8 and HOC7 cells were treated with indicated doses of AZD1775 and MKC8866 at various drug ratio. OD values at 450 wavelength was measured by CCK8 assay after drug administration for 48 h. Drug synergy analysis were performed by CompuSyn software 1.0 (ComboSyn Incorporated; http://www.combosyn.com).^[^ ^44^ ^]^ The combination effects were defined by combination index (CI) values with synergistic (<0.9), additive (0.9–1.1) and antagonistic (>1.1).

### Statistical Analysis

Statistical analysis and data plotting were performed using GraphPad Prism 8.0 or SPSS software. The unpaired two‐tailed Student's *t*‐test was performed when two groups were compared. For comparisons of multiple groups, one‐way ANOVA with Bonferroni post hoc test was used. Pearson's correlation coefficient was used to compute correlations between variables, using *t*‐test to assess the significance of the correlation. The data shown were the averages and standard deviations (SD) or standard error of the mean (SEM). Biological triplicates or duplicates from at least three independent experiments with similar results. *p* < 0.05 was considered statistically significant. In the figures, *, ** and *** refer to *p* < 0.05, *p* < 0.01, and *p* < 0.001, respectively.

## Conflict of Interest

Q.Z. is an employee and shareholder of Fosun Orinove. G.B.M. is a SAB (Scientific Advisory Board) member or consults with AstraZeneca, Chrysallis Biotechnology, GSK, ImmunoMET, Ionis, Lilly, PDX Pharmaceuticals, Signalchem Lifesciences, Symphogen, Tarveda, Turbine, Zentalis Pharmaceuticals. G.B.M. has stock options with Catena Pharmaceuticals, ImmunoMet, SignalChem, Tarveda, and has licensed technology HRD assay to Myriad Genetics, DSP patents with Nanostring.

## Author Contributions

R.X. and L.Y. contributed equally to this work. R.X. and L.Y. designed, performed, and analyzed experiments. L.Z., X.G., W.L., X.Y., L.Z., F.L., C.L., T.Q., and Y.C. performed and verified experiments. X.G., J.L., W.W., X.Z., and F.L. collected the data. E.G., Y.J., F.Z., B.Y., X.L., Y.F., Z.W., and W.P. analyzed RNA‐seq data and performed bioinformatics analysis. Q.Z., X.H., and B.W. helped perform the analysis with constructive discussions. R.X. and L.Y. wrote the manuscript. G.B.M., G.C., X.S., and C.S. reviewed and edited the manuscript. All authors read and approved the manuscript.

## Supporting information

Supporting Information

Supporting Table 1

## Data Availability

The data that support the findings of this study are available from the corresponding author upon reasonable request.
