# Supplementary material for: Inhibiting the IRE1α Axis of the Unfolded Protein Response Enhances the Antitumor Effect of AZD1775 in TP53 Mutant Ovarian Cancer
Source: Adv Sci (Weinh). 2022 May 26;9(21):2105469. doi: 10.1002/advs.202105469 (PMC9313493; doi:10.1002/advs.202105469)
Supplement: Supplementary file 1 — Supporting Information [file ADVS-9-2105469-s001.pdf]

ADVANCED SCIENCE
Open Access

Supporting Information

for *Adv. Sci.*, DOI 10.1002/advs.202105469

Inhibiting the IRE1α Axis of the Unfolded Protein Response Enhances the Antitumor Effect of AZD1775 in *TP53* Mutant Ovarian Cancer

Rourou Xiao, Lixin You, Li Zhang, Xichen Guo, Ensong Guo, Faming Zhao, Bin Yang, Xi Li, Yu Fu, Funian Lu, Zizhuo Wang, Chen Liu, Wenju Peng, Wenting Li, Xiaohang Yang, Yingyu Dou, Jingbo Liu, Wei Wang, Tianyu Qin, Yaoyuan Cui, Xiaoxiao Zhang, Fuxia Li, Yang Jin, Qingping Zeng, Beibei Wang, Gordon B. Mills, Gang Chen, Xia Sheng* and Chaoyang Sun**

Figures S1

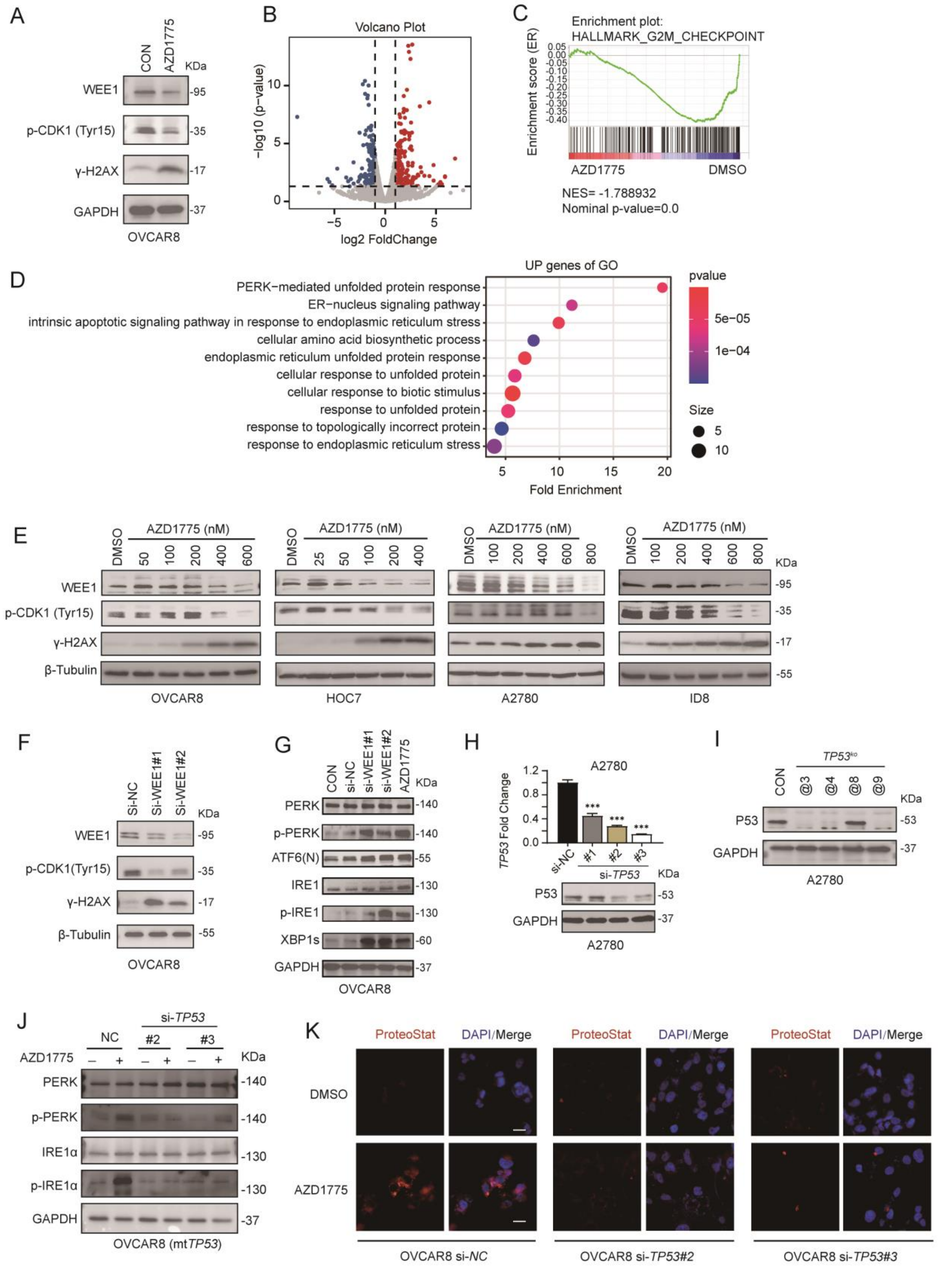

Figures S1. AZD1775 triggers UPR in *TP53* mutant ovarian cancer cells. **A)** The Protein levels of WEE1, p-CDK1 (Tyr15) and γ-H2AX of OVCAR8 were determined by Western analysis after treated with or without 400 nM AZD1775 for 72 hours. **B)** Volcano plot of

differential genes on the RNA-seq data of OVCAR8 cells treated with 400 nM AZD1775 for 48 hours determined by *P* value. **C)** GSEA plot of the Hallmark pathway G2M checkpoint enriched by GSEA on the RNA-seq data of OVCAR8 treated with 400 nM AZD1775 for 48 hours. **D)** Pathways enriched by GO analysis on the RNA-seq data of OVCAR8 cells treated with 400 nM AZD1775 for 48 hours. **E)** the Protein levels of WEE1, p-CDK1 (Tyr15) and γ-H2AX of ovarian cancer cell lines were determined by Western analysis after treated with a series of indicated does of AZD1775 for 72 hours. 400 nM AZD1775 was used for further study. **F)** Western blot assay of the Protein levels of WEE1, p-CDK1 (Tyr15) and γ-H2AX. OVCAR8 were transfected with scramble siRNA (si-NC) or two *WEE1* siRNAs. **G)** OVCAR8 cells were transfected with scramble siRNA (si-NC) or two WEE1 siRNAs and the expression level of key proteins in the UPR was determined by Western blot. AZD1775 was used as a positive control. **H)** qPCR and Western blot analysis of gene silencing by siRNAs against *TP53* for 48 hours in A2780. The expression of scramble siRNA(si-NC) was used as control. Data across panels represent mean ± SEM of three independent experiments. ***$p < 0.001$, as determined by unpaired t test. **I)** Western blot assay of p53 expression in CRISPR-Cas9 mediated $TP53^{ko}$ clones of A2780 cells. **J)** Western blot analysis of the protein expression levels of PERK, p-PERK, IRE1α, p-IRE1α. OVCAR8 cells were treated with or without 400 nM AZD1775 for 72 hours after transfected with scramble siRNA (NC) or *TP53* siRNA. #2 and #3 denote different *TP53* siRNAs. **K)** Representative images of aggregated proteins. OVCAR8 cells were treated with or without 400 nM AZD1775 for 72 hours after transfected with scramble siRNA (NC) or *TP53* siRNA. #2 and #3 denote different *TP53* siRNAs and stained with Proteostat Dye. Scale bar, 20 µm.

Figures S2

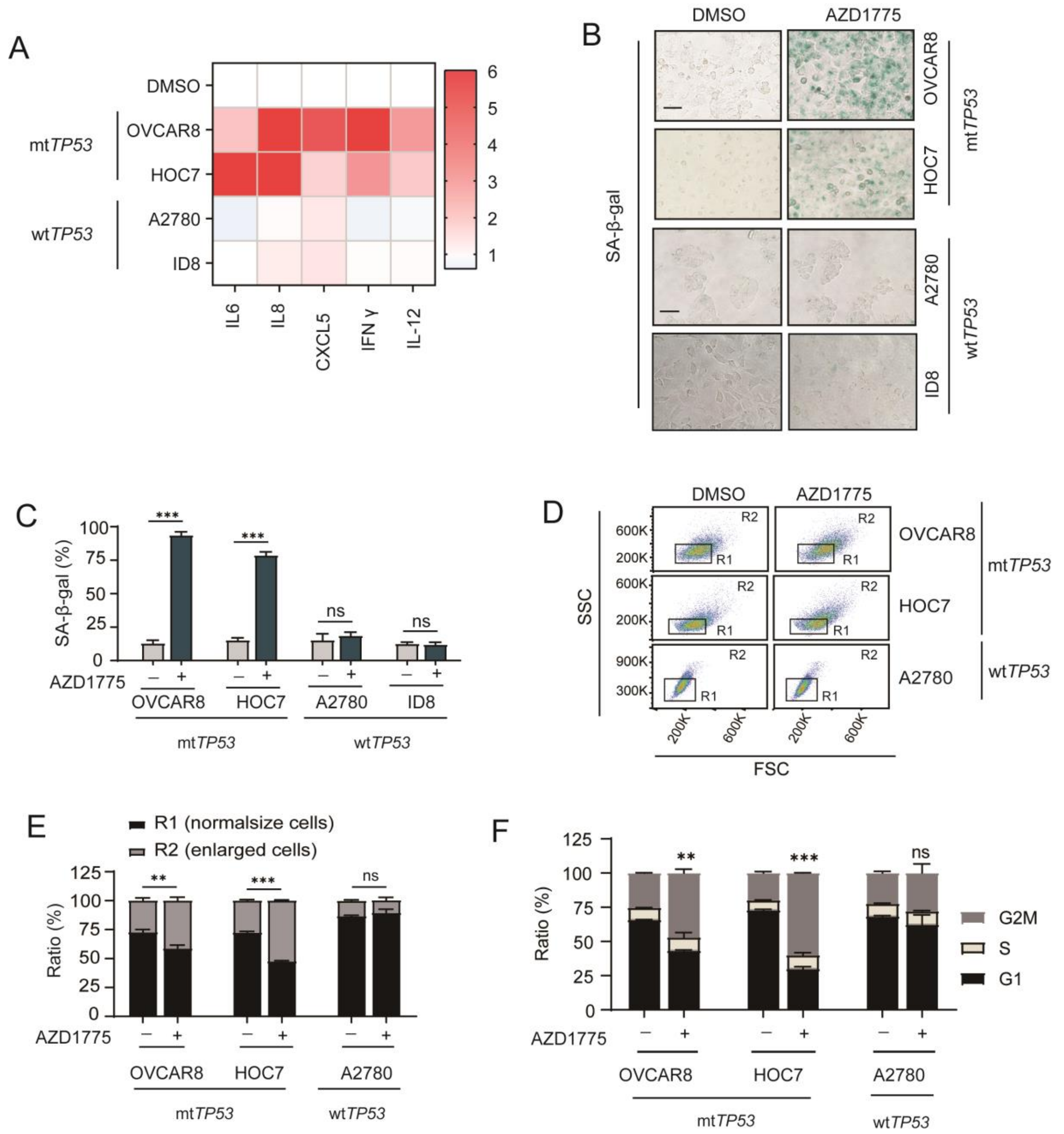

Figures S2. AZD1775 induces senescence in *TP53* mutant ovarian cancer cells. **A)** RT-qPCR was used to examine the mRNA levels of genes IL-6, IL-8, CXCL5, IFN γ and IL-12. The relative mRNA expression of these genes were shown as heatmap. The color scale denotes the mean of fold change (n = 2). Cells were treated with DMSO or 400 nM AZD1775 for 48 hours. **B)** Representative SA-β-gal staining image of ovarian cancer cell lines after treated with DMSO or 400 nM AZD1775 for 72 hours. The data represent three independent experiments. Scale bar, 50 μm. **C)** Quantification of SA-β-gal positive cells in **(B)**. Error bars represent the SD of the mean (n = 3). ***p < 0.001, ns, not significant, as determined by unpaired two-tailed Student's t test. **D)** Representative morphology by flow cytometry

analysis of the cell population plot for forward scatter factor (FSC, indicative of size, X-axis) and side scatter factor (SSC, indicative of granularity, Y-axis) (R2 =high FSC and SSC; R1 = normal FSC and SSC) after 400nM AZD1775 for 48 hours. **E)** Quantification of normal sized cells and enlarged cells. Error bars represent the SD of the mean count of R2 (n = 3). **p < 0.01, ***p < 0.001, ns, not significant, as determined by unpaired two-tailed Student's t test. **F)** Quantification of the cell cycle populations of flow cytometry analysis of ovarian cancer cells following 48 hours exposure to 400nM AZD1775. Error bars represent the SD of the mean count of cells in G2M phase (n = 3). **p < 0.01, ***p < 0.001, ns, not significant, as determined by unpaired two-tailed Student's t test.

Figures S3

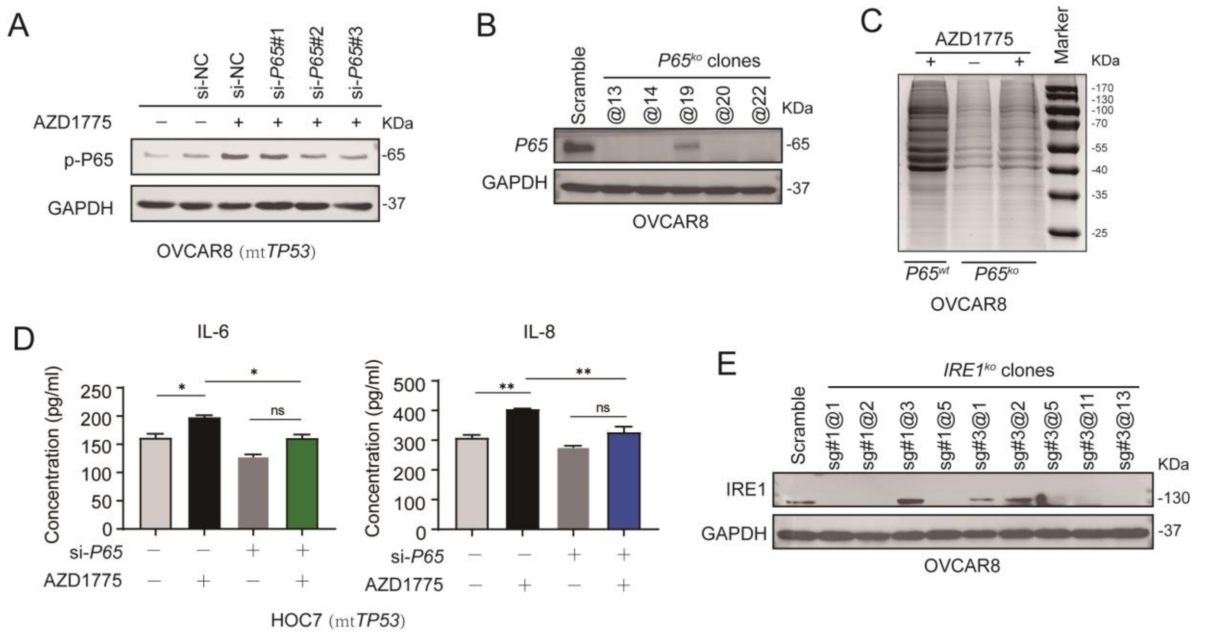

Figures S3. AZD1775-triggered UPR was induced via NF-κB-dependent SASP in *mtTP53* ovarian cancer cells. **A)** Western blot assay of gene silencing by siRNAs against p65 for 24 hours and incubation with or without 400 nM AZD1775 for 24 hours before harvested. **B)** Western blot assay of gene silencing by CRISPR-Cas9 technology mediated *P65*-knockout ($P65^{ko}$) in OVCAR8 cells. $P65^{ko}$ clone@13 was selected for further study. **C)** Intracellular soluble protein assay of OVCAR8 cells treated with 400 nM AZD1775 before or after $P65^{ko}$. **D)** Levels of IL-6 and IL-8 in cell culture supernatant were measured by ELISA assay. HOC7 cells were transfected with si-NC or p65 siRNA#3 (si-*p65*) verified in **(A)** and treated with or without 400 nM AZD1775 for 24 h. Error bars denote the SD, *$p < 0.05$, **$p < 0.01$, ns, not significant, as determined by ANOVA with Bonferroni post hoc test. **E)** Western blot assay showing gene silencing by CRISPR-Cas9 technology mediated *IRE1*-knockout ($IRE1^{ko}$) in OVCAR8 cells. $IRE1^{ko}$clone@1 was selected for further study.

Figures S4

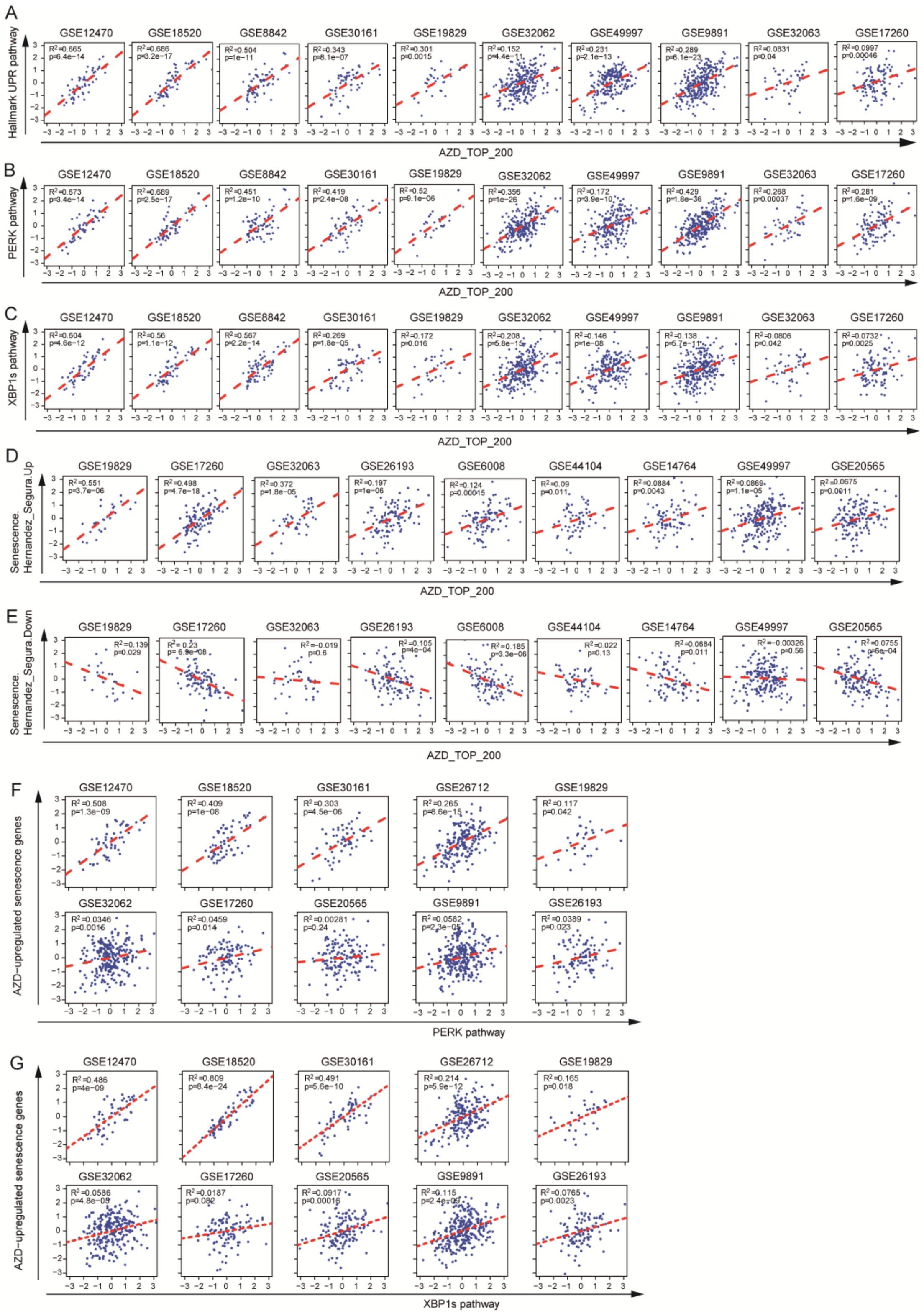

Figures S4. AZD1775 induced signature, senescence, and UPR are functionally related in clinical datasets of ovarian cancer. **A-G**) The rest of each individual correlation plots as in Figure 3.

Figures S5

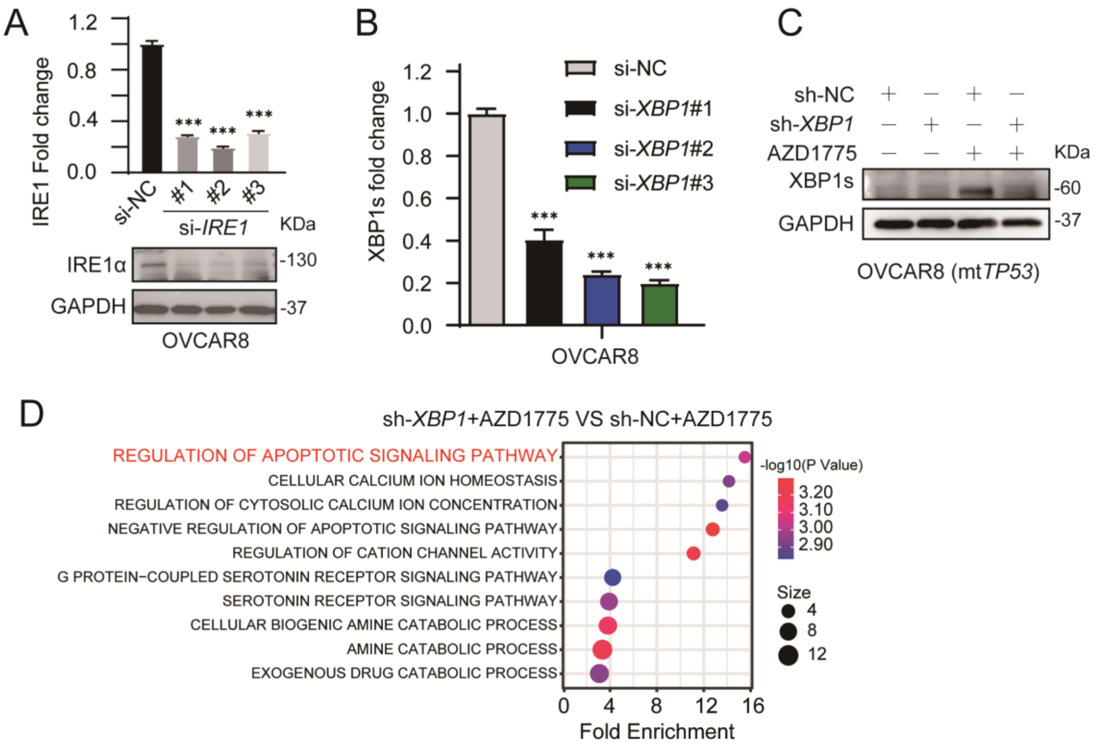

Figures S5. Inhibition of IRE1α-XBP1 branch promoted CHOP-dependent apoptosis. **A, B)** Western blot assay and qPCR analysis of gene silencing by siRNA against *IRE1* or *XBP1* for 48 hours in OVCAR8. The expression of si-NC was used as control. data represent three independent experiments. Error bars represent the SD of the mean (n = 2). ***p < 0.001, as determined by unpaired two-tailed Student's t test. **C)** Western blot analysis of XBP1s expression in OVCAR8 cells transduced with sh-NC or sh-XBP1 and incubated with or without 400nM AZD1775 for 72 hours. **D)** Pathways enriched by GO analysis on the RNA-seq data of OVCAR8 cells transduced with sh-NC or sh-XBP1 and incubated with or without 400nM AZD1775 for 72 hours.

Figures S6

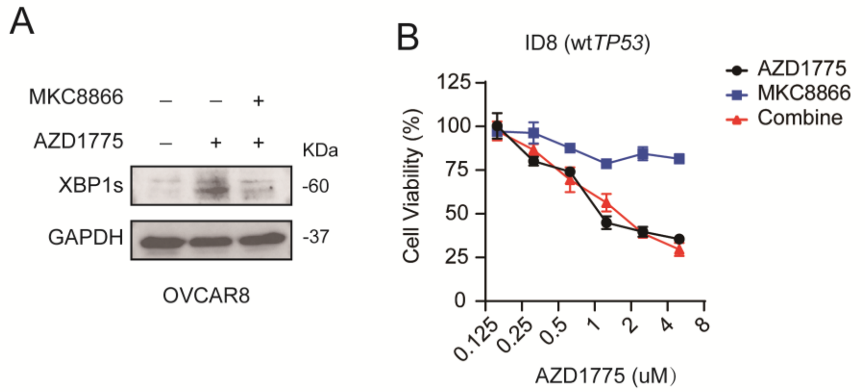

Figures S6. AZD1775 and MKC8866 were not synergistic in ID8 cells. A) Western blot analysis of XBP1s expression in OVCAR8 cells treated with or without MKC8866 at a concentration of 10 μM and incubated with or without 400 nM AZD1775 for 72 hours. **B)** Cell viability of ID8 was measured by CCK8 after treated with a series of indicated doses of AZD1775 and MKC8866 at specified ratios of 1:2.5 for 48 hours. Biological triplicates from one representative experiment of two performed with similar results.
